# Inflation nach Corona: Sind die Sorgen berechtigt?

**DOI:** 10.1007/s10273-021-2996-0

**Published:** 2021-09-21

**Authors:** ZG Überblick

**Keywords:** E31, E50, E52

## Abstract

Im August 2021 wird die deutsche Inflationsrate voraussichtlich auf 3,9 % steigen. Ein Teil davon dürfte einmaligen oder vorübergehenden Effekten geschuldet sein. Hierzu zählen in Deutschland vor allem die Rücknahme der Mehrwertsteuersenkung, die Ausweitung der CO_2_-Bepreisung sowie Preisanstiege durch die Knappheit von Rohstoffen und Vorprodukten. Dennoch mag es strukturelle Gründe für eine steigende Inflation geben. So werden zum Teil die lockere Geldpolitik und das neue Inflationsziel der EZB für höhere Inflationsrisiken verantwortlich gemacht. Außerdem führt die expansive Fiskalpolitik der neuen US-Administration zu höheren Preissteigerungsraten in den USA. Ob die Möglichkeit einer Lohn-Preis-Spirale besteht und ob die Inflationsmessung verbesserungsfähig ist, wird ebenfalls im Rahmen dieses Zeitgesprächs diskutiert.

## Abstract (en)

In August 2021, the German inflation rate is expected to rise to 3.9 %. Part of this is likely due to one-off effects. In Germany, these include above all the reversal of the VAT cut, the expansion of CO_2_ pricing and price increases due to the scarcity of raw materials and intermediate products. Nevertheless, there may also be structural reasons for rising inflation. For example, the loose monetary policy and the ECB’s new inflation target are partly to blame for higher inflation risks. In addition, the expansive fiscal policy of the new Biden administration is leading to higher rates of price increases in the US. Other aspects, the possibility of a wage-price spiral and whether inflation is measured correctly at all, will also be discussed in this Zeitgespräch.

